# ALPPS Procedure for Extended Liver Resections: A Single Centre Experience and a Systematic Review

**DOI:** 10.1371/journal.pone.0144019

**Published:** 2015-12-23

**Authors:** Marco Vivarelli, Paolo Vincenzi, Roberto Montalti, Giammarco Fava, Marcello Tavio, Martina Coletta, Andrea Vecchi, Daniele Nicolini, Andrea Agostini, Emad Ali Ahmed, Andrea Giovagnoni, Federico Mocchegiani

**Affiliations:** 1 Hepatobiliary and Abdominal Transplantation Surgery, Department of Experimental and Clinical Medicine, Polytechnic University of Marche, Ancona, Italy; 2 Department of Gastroenterology, Polytechnic University of Marche, Ancona, Italy; 3 Unit of Emerging and Immunosuppressed Infectious Diseases, Department of Gastroenterology and Transplantation, Polytechnic University of Marche, Ancona, Italy; 4 Unit of General and Paediatric Radiology, Department of Radiology, Polytechnic University of Marche, Ancona, Italy; 5 Hepatobiliary and Pancreatic Surgery Unit, Department of General Surgery, Faculty of Medicine, Sohag University, Sohag, Egypt; University Hospital Oldenburg, GERMANY

## Abstract

**Aim:**

To report a single-centre experience with the novel Associating Liver Partition and Portal vein ligation for Staged hepatectomy (ALPPS) technique and systematically review the related literature.

**Methods:**

Since January 2013, patients with extended primary or secondary liver tumors whose future liver remnant (FLR) was considered too small to allow hepatic resection were prospectively assessed for the ALPPS procedure. A systematic literature search was performed using PubMed, Scopus and the Cochrane Library Central.

**Results:**

Until July 2014 ALPPS was completed in 9 patients whose mean age was 60±8 years. Indications for surgical resection were metastases from colorectal cancer in 3 cases, perihilar cholangiocarcinoma in 3 cases, intrahepatic cholangiocarcinoma in 2 cases and hepatocellular carcinoma without chronic liver disease in 1 case. The calculated FLR volume was 289±122 mL (21.1±5.5%) before ALPPS-1 and 528±121 mL (32.2±5.7%) before ALLPS-2 (p<0.001). The increase in FLR between the two procedures was 96±47% (range: 24–160%, p<0.001). Additional interventions were performed in 4 cases: 3 patients underwent Roux-en-Y hepaticojejunostomy, and one case underwent wedge resection of a residual tumor in the FLR. The average time between the first and second step of the procedure was 10.8±2.9 days. The average hospital stay was 24.1±13.3 days. There was 1 postoperative death due to hepatic failure in the oldest patient of this series who had a perihilar cholangiocarcinoma and concomitant liver fibrosis; 11 complications occurred in 6 patients, 4 of whom had grade III or above disease. After a mean follow-up of 17.1±8.5 months, the overall survival was 89% at 3–6 and 12 months. The recurrence-free survival was 100%, 87.5% and 75% at 3-6-12 months respectively. The literature search yielded 148 articles, of which 22 articles published between 2012 and 2015 were included in this systematic review.

**Conclusion:**

The ALPPS technique effectively increased the resectability of otherwise inoperable liver tumors. The postoperative morbidity in our series was high in accordance with the data from the systematic review. Age, liver fibrosis and presence of biliary stenting were predisposing factors for postoperative morbidity and mortality.

## Introduction

Surgical resection is a potentially curative treatment for patients with primary and secondary malignant liver tumors. Although the liver has the unique capability to regenerate, a remnant of at least 20% of the liver volume must be spared to avoid postoperative liver failure provided that the parenchyma not affected by the tumor is anatomically normal. In the presence of chemotherapy-induced liver injury, the future liver remnant (FLR) should be at least 30% of the total volume; however, in the presence of cirrhosis, a 40% FLR is advisable[1].

Strategies have been developed to increase the resectability of those tumors that are too advanced to be resected leaving a sufficient FLR. These strategies, namely right portal embolization (PVE) and preoperative or intraoperative ligation of the right portal vein (PVL), are based on the occlusion of the flow in one of the main branches of the portal vein (PVO) inducing atrophy in the ipsilateral liver and subsequent hypertrophy of the contralateral lobe; due to the larger volume of the right liver, usually the right branch of the portal vein is occluded to increase the volume of the left liver. In fact, the occlusion of the right portal vein induces compensatory hypertrophy of the left lobe that is on the average of 40% in approximately 4–8 weeks[2]. However, in the case of fast-growing tumors, the time required to obtain compensatory hypertrophy is often too long to ensure the operability, and the degree of the compensatory hypertrophy is often lower than expected[3].

Recently, a new technique of hepatic resection that is performed in two stages—called Associating Liver Partition and Portal vein ligation for Staged hepatectomy (ALPPS)—has been described. The first step of this procedure associates the intraoperative ligation of the right portal branch to the partition of the liver, usually following the scheme of an extended right hepatectomy. In contrast to a classical hepatectomy, the diseased part of liver is left in situ and remains vascularized by the right hepatic artery only, while the biliary and systemic venous drainage through the right biliary duct and hepatic veins, respectively, are preserved[4]. In the second step of the procedure that is usually performed within 7 to 15 days after the first, the diseased part of the liver is removed by simply sectioning the remaining biliary, hepatic arterial and systemic venous pedicles. This innovative procedure allows rapid and significant hypertrophy of the FLR, thus ensuring a wider operability than previous techniques.

The purpose of this study was to describe our initial experience with extended two-stage liver resections using the ALPPS technique for the treatment of primary or metastatic liver tumors. A systematic review of the literature concerning the ALPPS procedure was also carried out in order to highlight the differences in indications, surgical techniques, increase in FLR and clinical outcome.

## Materials and Methods

From February 2005 to September 2014, 553 patients underwent liver resection at the Hepatobiliary and Abdominal Transplantation Surgery of the Polytechnic University of Marche, Ancona, Italy. All patients were evaluated for liver surgery in a multidisciplinary meeting attended by surgeons, radiologists, interventional radiologists and gastroenterologists. Evaluation of the FLR (%) was performed in all cases of planned right extended hepatectomy and in those cases where a right hepatectomy was planned in patients with liver disease. Before January 2013, an FLR <30% was considered a contraindication for surgical resection and an indication for right preoperative portal vein embolization followed by CT after a mean time of 5 weeks. Surgical resection was then performed only when an increase in the FLR to values >30% was observed after PVE. Patients who did not develop sufficient hypertrophy or who showed tumor progression were excluded from surgery and received palliative treatment. Since January 2013, all patients with an FLR <30% were assessed for the ALPPS procedure.

Exclusion criteria for the ALPPS procedure were as follows: the presence of distant metastases, age >75 years, chronic liver disease and macrovesicular steatosis greater than 50%. In all cases with diagnosed or suspected liver disease, a liver biopsy and/or the indocyanine green test measured with spectrophotometry were performed preoperatively.

The data of patients undergoing ALPPS were prospectively collected. All study enrollment procedures and subsequent data collection and acquisition were approved by the Institutional Review Board at the Ospedali Riuniti Hospital. Written informed consent was obtained from the patients. Postoperative complications were classified according to the Dindo-Clavien classification[5].

### Liver Volumetric Estimation

The future liver remnant (FLR) was estimated as a fraction of FLR (mL) and the total functioning liver, the latter being the difference between the total liver volume (TLV) and tumor volume (TuV)[6].

$$FLR\;(\%)=100\;\times \;\frac{FRLV\;(cc)}{TLV\;[{cm}^{3}]-TuV\;[{cm}^{3}]}$$

CT scans were conducted using a 64-row scanner (GE LightSpeed VCT 64). All images were obtained at 120 kV and automated mA. A pre-contrast acquisition and post-contrast tri-phasic protocol were used. After the administration of 1.5 ml/kg body weight of 370 mg/ml iodine contrast material, an arterial phase (bolus tracking: 150-HU threshold in abdominal aorta, 7-s delay), a portal venous phase (25–35 s from the arterial phase) and a late venous phase (25–35 s after the portal venous phase) were obtained. All volumetric acquisitions were reconstructed in contiguous axial slices of 2.5 mm.

MR scans were performed using a superconductive high-field scanner (GE HdXt 1.5T). For volumetric evaluation, contrast-enhanced breath-hold 3D GRE T1w images were used, obtained after the administration of 0.1 mmol/kg body weight of Gd-DOTA or Gd-EOB-DTPA 0.025 mmol/kg body weight. An arterial phase (15–20 sec after bolus injection), a portal phase (45–55 s after bolus injection), a late venous phase (90–100 sec after bolus injection) and a hepatobiliary phase at 20 min (after Gd-EOB-DTPA) were obtained. All processed axial images had a slice thickness of 4 mm and a slice spacing of 2 mm[7].

Volumetric evaluation was performed using an Advantage Workstation 4.4 (GE Medical Systems, Milwaukee, USA). The manual contouring technique on a single axial slice was used, with the total volume calculated as the sum of voxels within areas manually defined. Couinaud’s vascular landmarks were used for virtual resections[8].

**Fig 1:**
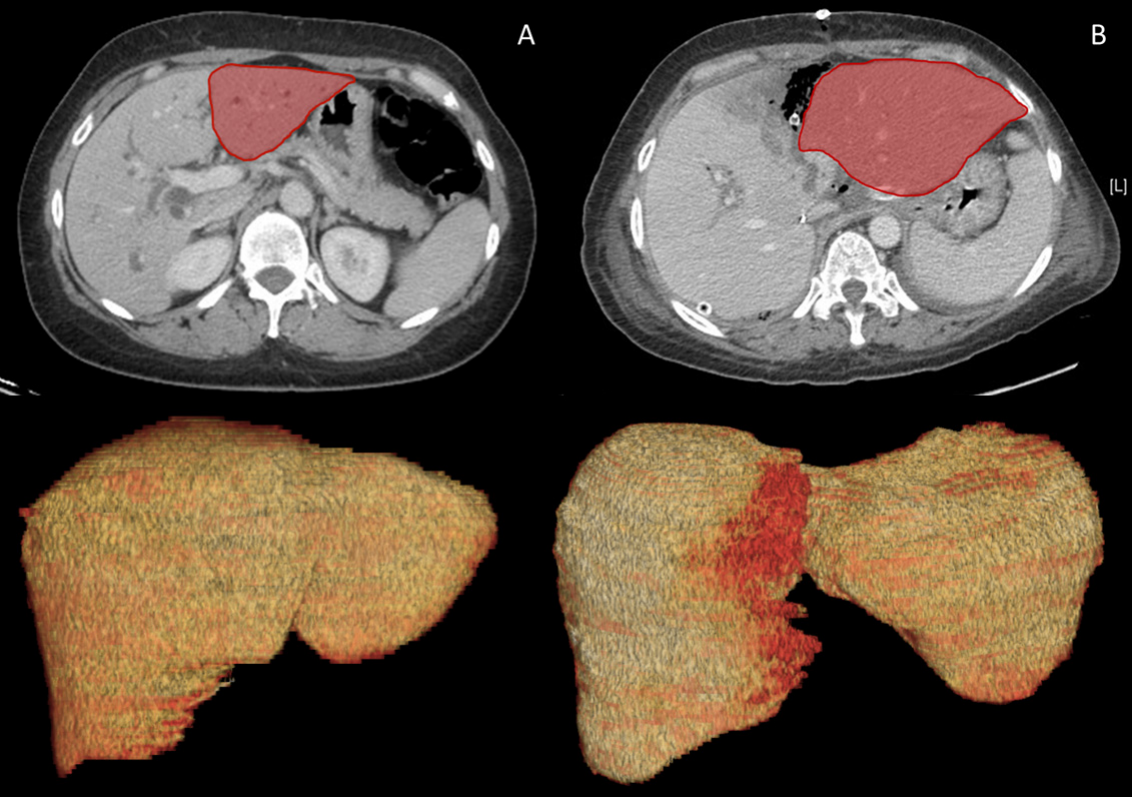
Case #1. FLR before ALPPS-1 (A) and after 6 days (B) in a patient with pCCA.

All volumes were estimated subtracting portal pedicles until segmental branches, main hepatic trunks, and biliary tree when obstructive jaundice was detected (Fig 1). Tumor volume was calculated only for mass-forming intrahepatic tumors and not for perihilar cholangiocarcinoma. To reduce the risk of errors, each measurement is the mean of multiple simulations.

### Surgical Technique

#### First Step of ALPPS (ALPPS-1)

In all cases, we performed a right subcostal abdominal incision with midline extension. After exploration of the peritoneum, intraoperative ultrasound examination was performed to evaluate the characteristics of the neoplastic nodules and their relationship with the vascular and biliary tree. Hilar lymph node dissection was performed with extemporaneous histological examination. The right liver was detached from the vena cava by ligating and dividing the accessory hepatic veins. The following elements were identified: common bile duct, right and left hepatic artery, right branch of the portal vein, portal vein branch for segment 4 and right hepatic vein. The right portal branch and PV branch for segment 4 were isolated and ligated. Differently from what was described in the first report of the ALPPS technique, the middle hepatic vein was always preserved at step 1, anticipating a recommendation that was subsequently given in a consensus conference[9].

In the case of small lesions in the FLR, these were removed with wedge resections.

Liver parenchyma transection was performed using an electric scalpel, the Ligasure and/or Sonoca (Söring GmbH). Liver transection was performed up to the anterior surface of the vena cava.

**Fig 2:**
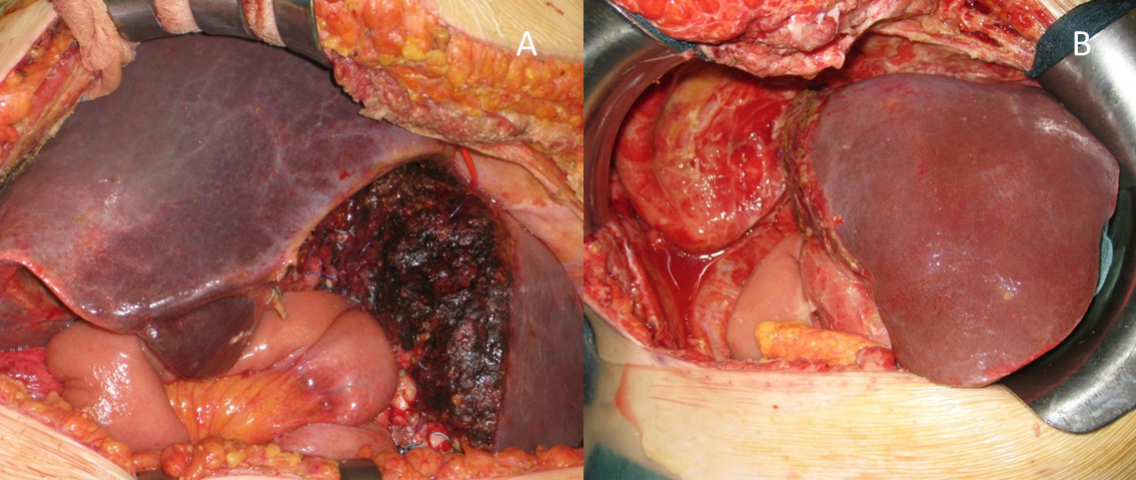
Case #1. Intraoperative picture at the end of first step of ALPPS (A) and at the end of second step of ALPPS (B). Roux-en-Y hepaticojejunostomy was performed during this first step of ALPPS.

At the end of the hepatic transection, the two cutting surfaces of the liver were separated using an organ bag or a plastic sheet or sheets of Tachosil. In the presence of extrahepatic biliary tumors, a Roux-en-Y hepaticojejunostomy was performed during this first step of the ALPPS (Fig 2A). Two abdominal drains were placed.

The right hepatic vein, right hepatic artery, hepatic artery for segment 4 and right branch of the bile duct were surrounded with vessel loops fixed with titanium clips to allow better identification of these structures at the second step of the procedure.

All patients were administered parenteral therapy support and antibiotic therapy. A CT scan or abdominal MRI with volumetric reconstruction was performed to 7±2 days after the first operation. When the FLR increased to at least 30% of the liver volume, the second step of the ALPPS was indicated and performed the following day. When the FLR was lower than 30% of the liver volume, and the patient was in stable clinical conditions, the second step of the ALPPS was delayed.

#### Second Step of the ALPPS (ALPPS-2)

A re-laparotomy was performed on the previous incision line, and the liver was freed from adhesions. The right hepatic vein, right hepatic artery, right branch of the bile duct, and arterial branch to segment 4 were identified, ligated and divided; right trisectionectomy was then completed (Fig 2B).

### Postoperative Management and Follow-Up

Antibiotic prophylaxis and parenteral-enteral nutrition was initiated after the first step and maintained during the first three days after the second step. Deep venous thrombosis prophylaxis was utilized in all patients[10].

Post-hepatectomy liver failure was defined according to 50–50 criteria (prothrombin time <50% and total serum bilirubin >50 mmol/L on postoperative day 5 or after)[11].

After discharge, patients were referred to medical oncologists for the follow-up that included abdominal ultrasound and abdominal CT-scan or MRI 3 and 6–12 months after surgery, respectively. Serum tumor markers were assessed at 3-6-12 months after surgery.

### Systematic Review

#### Literature Search

PRISMA statement guidelines to conduct and report systematic reviews were followed[12]. The research protocol was registered at the International Prospective Register of Systematic Reviews (http://www.crd.york.ac.uk/PROSPERO) with the following registration number: CRD42014014563.

A systematic literature search was performed independently by two of the authors (RM and DN) using PubMed, Scopus and the Cochrane Library Central. The search was limited to humans and articles reported in the English language. No restriction was set regarding the type of publication, date or publication status. Participants of any age and sex who underwent ALPPS procedures were considered. The search strategy was based on different combinations of words for each database. For the PubMed database, the following combination was used: *“ALPPS” OR “associating liver partition and portal vein ligation for staged hepatectomy” OR “ALTPS” OR “right portal vein ligation” OR”portal vein transection” OR “right portal occlusion” OR "in situ liver transection"*.

The same key words were inserted in the search manager fields of Scopus and the Cochrane Library Central. The search was broadened by extensive cross-checking of reference lists of all retrieved articles fulfilling inclusion criteria. For all databases, the last search was run on April 23, 2015.

#### Study Selection

The same two authors independently screened the title and abstract of the primary studies that were identified in the electronic search. Duplicate studies were excluded. The following inclusion criteria were set for inclusion in this systematic review: 1) studies describing ALPPS procedures; 2) studies reporting at least one perioperative outcome; 3) if more than one study was reported by the same institute, only the most recent or the highest quality study was included.

The following exclusion criteria were set: 1) review articles, letters, comments and case reports; and 2) studies where it was impossible to retrieve or calculate data of interest.

#### Data Extraction

The same two authors extracted the following main data: 1) first author, year of publication and study type; 2) number and characteristics of patients; 3) increase of FLR between stage 1 and 2, rate of patients who failed to perform ALPPS-2, time (days) between stage 1 and 2, R0 margins (%), indication for ALPPS, overall and severe complications, tumor recurrence up to 12 months, surgical time (minutes), variations to the original technique, recipient morbidity, and in-hospital mortality. Bias of the individual studies was categorized based on study design. All relevant texts, tables and figures were reviewed for data extraction. Discrepancies between the two reviewers were resolved by consensus discussion.

#### Statistical Analysis

Continuous variables normally distributed were described as means±SD, while non-normally distributed variables were described as medians (range). A comparison of pre- and post-liver volumes of ALPPS 1 was performed using Student's t test for paired data. Survival analysis was calculated using the analysis of life tables. Statistical analysis was performed using IBM® SPSS® Statistics ver. 19.0 software.

## Results

### Preoperative

From January 2013 to June 2014, the ALPPS procedure was indicated in 11 cases. Two patients were judged intraoperatively not to be suitable for the procedure and, therefore, are not included in the present analysis: these 2 patients underwent surgery due to perihilar cholangiocarcinoma and were excluded from the planned ALPPS procedure because of severe liver fibrosis diagnosed by intraoperative liver biopsy in one case, and because of intraoperative evidence of a hilar lymph node metastases in the other. The main characteristics of the 9 patients who underwent the ALPPS procedure are described in Table 1.

**Table 1:** Preoperative patients and tumor characteristics.

**Sex (M/F)**	2/7
**Age (years)**	60±8
**BMI (Kg/m2)**	**26.1±3.6**
**Diagnosis**	
CRLM	3
pCCA	3
iCCA	2
HCC	1
**Comorbidity**	
Cardiovascular	3
Pulmonary	1
Diabetes	0
**Previous abdominal surgery**	6
**α-FP (HCC, ng/dl)** *	6.7
**CEA (CRLM, ng/dl)**	37 (6.8–386)
**CA 19–9 (Biliary tumors, ng/dl)**	7 (5–54)

CRLM: colorectal liver metastases; iCCA: intrahepatic cholangiocarcinoma; pCCA: perihilar cholangiocarcinoma; HCC: hepatocellular carcinoma.

*only one patient.

Perihilar cholangiocarcinoma (pCCA) was the indication for ALPPS in 3 patients of whom 2 had type IV and 1 type IIIa tumor according with the Bismuth–Corlette classification. Two of the 3 patients with pCCA had a biliary stent placed endoscopically before surgery to relieve jaundice.

Six patients had undergone previous abdominal surgery, namely resection of colorectal cancer (4 patients), appendectomy and cholecystectomy (1 patient), and wedge hepatic resection for intrahepatic cholangiocarcinoma (1 patient).

### Liver Volumetry

Table 2 and S1 Dataset describe the characteristics of the liver volumes before and after the first step of ALPPS. The volumetric liver study was performed using CT or MRI.

**Table 2:** Liver volumetry pre- and post-first step of ALPPS.

	Before ALPPS-1	After ALPPS-2	p
**Imaging**			
CT-scan	5	8	NA
MRI	4	1	NA
**Total liver volume (mL)**	1336 (943–4205)	1621 (1044–5071)	0.008
**Tumor volume (mL)** *	199 (17–2089)	178 (31–2211)	0.406
**FLR (mL)**	289±122	528±121	<0.001
**FLR (%)**	21.1±5.5	32.2±5.7	<0.001
**Increase of FLR (mL)**	239±88		
**Increase of FLR (%)**	96%±47		

*not calculated for pCCA tumors.

**Fig 3:**
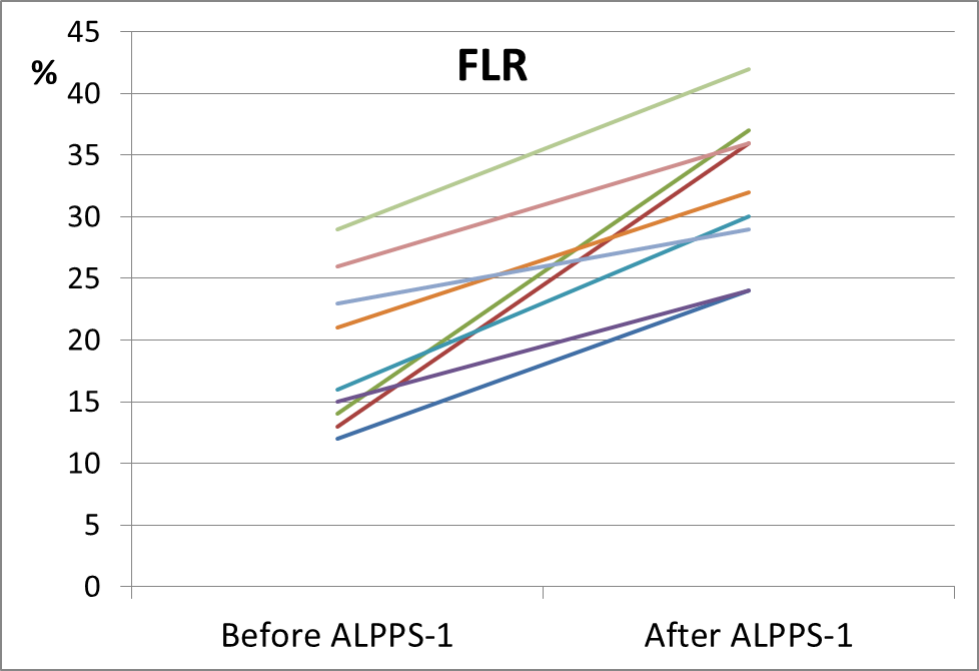
FLR increase between the ALPSS-1 and ALPPS-2 procedures (7.4±2 days).

The liver volumes were calculated before the first surgical step and after 7.4±2 days (Fig 3).

The volumetric measurement before ALPPS-1 was based on MRI in 5 cases and on CT in 4 cases. The volumetric study before ALPPS-2 was based on CT in 8 cases and MRI in 1 case (due to impairment of the renal function).

### Intraoperative Data

Table 3 summarizes the main aspects of the first surgery.

**Table 3:** Intraoperative data of the first step of ALPPS.

**Surgical time (min)**	429±113
**FLR (segments)**	
Segments 1-2-3	4
Segments 2–3	4
Segments 1-2-3-4b	1
**Blood transfusion**	5
PRBC (cc)	550±270
**Pringle manoeuvre**	3
Timing (min)	8.33±2.88
**Concomitant operation (N° pts)**	8
Roux-en-Y hepaticojejunostomy	3
Wedge left lobe	1
Cholecystectomy	5
**Material between surgical surface**	
Plastic bag	1
Plastic sheet	5
Tachosil	3

In 5 cases, the liver transection was set for right trisectoniectomy; in 4 cases, the resection was extended to segment I. In one patient, it was possible to preserve segment IVb; in this latter case the middle hepatic vein was spared while in all the other patients the middle hepatic vein was ligated and divided, always at the second step of the operation.

Five patients required blood transfusions, with a mean required volume of 650±319 cc. No plasma or platelet transfusions were required.

Intermittent hilar clamping was performed in 3 patients, with a total duration of 8.33±2.88 minutes.

Additional interventions were performed in 4 cases: 3 patients underwent Roux-en-Y hepaticojejunostomy; in one case, a wedge resection of a residual tumor in the FLR was carried out. In all cases, a cholecystectomy was performed (if not done previously).

In the first case performed, an organ bag was used to wrap the right liver and separate the two hemi-livers; in the following 5 cases, we used a sheet of plastic material; in the last three cases, we employed sheets of haemostatic TachoSil.

In all patients, it was possible to perform the second step. The second surgery had a surgical time of 198±59 min. Blood transfusions were required in 5 patients with an average volume of 400±141 cc. No plasma or platelets transfusions were required; hilar clamping was not performed.

### Histology

Table 4 summarizes the main histological features of the tumors.

**Table 4:** Pathology.

**Diagnosis**	
CRLM	4
pCCA	3
HCC	1
iCCA	1
**N° nodules** *	2.2±1.5
**Diameter of the largest tumor (cm)** *	7.6±4.4
**Total tumor diameter (cm)** *	9.9±2.9
**R0**	9
**Liver steatosis**	3
Microvesicular	2
Macrovesicular	3
**Hepatic fibrosis**	1
**Lymph Nodes (pos)**	1

CRLM: colorectal liver metastases; iCCA: intrahepatic cholangiocarcinoma; pCCA: perihilar cholangiocarcinoma; HCC: hepatocellular carcinoma.

* Not calculated for pCCA.

Pathology confirmed the preoperative diagnosis in 8 cases; however, in one case where the preoperative diagnosis was intrahepatic cholangiocarcinoma, metastatic colorectal cancer was revealed. A tumor-free surgical margin was histologically confirmed in all cases (R0 resection = 100%). An average of 4.2 lymph nodes per patient was examined, and one case of intrahepatic cholangiocarcinoma was detected as neoplastic involvement.

### Postoperative course

There were no intraoperative deaths. The main postoperative results are described in Table 5.

**Table 5:** Postoperative data.

**Timing between ALPPS-1/ALPPS-2 (days)**	10.8±2.9
**Timing between ALPPS-1 and volumetry (days)**	7.4±2
**Patients with complications**	6
Biliary leak	3
Pulmonary embolism	2
Sepsis	2
Bleeding	2
Post-hepatectomy liver failure	1
Severe cytolysis	1
Infection	1
**Dindo-Clavien classification**	
1	1
2	1
3	1
4	2
5	1
**In-hospital mortality**	1
**Hospital stay (days)**	24.1±13.3
**1-year mortality**	1
**Causes of mortality**	
Liver failure	1
**Follow-up (months)**	17.1±8.5

Eight patients were kept in the hospital in the interval between the two operations, while one patient was temporarily discharged on postoperative day (POD) 6.

Overall, 11 complications occurred in 6 patients, 4 of which were grade III or above according to the Dindo-Clavien classification. There was one postoperative death due to liver failure (patient 6). In the latter patient, a 73-year-old woman with type IV perihilar cholangiocarcinoma, two weeks before ALPPS-1, an endoscopic internal biliary stent had been placed due to serum bilirubin >10 mg/dL, the FLR (mL) was 192 mL, and the FLR was 16%; at surgery, liver histology showed micro and macrovesicular steatosis of 10%. After ALPPS-1, the patient developed a biliary leak and sepsis caused by multiresistant *Klebsiella pneumoniae* and *Enterococcum faecium*. To try to control the abdominal sepsis, the second step of ALPPS was carried out 8 days after the first, although the increase of the liver volume was not completely satisfactory (FLR = 24%), and the patient developed postoperative liver failure and died on POD 21. Remnant liver biopsy revealed moderate cholestasis, mild steatosis and fibrosis.

Intraabdominal sepsis developed after ALPPS-1 in both patients who had endoscopically-placed biliary stent at the time of surgery, while it was observed only in 1 of the other 7 patients with no biliary stent.

Biliary leak was the most frequent postoperative complication that developed in 3 patients. The high-grade leaks (2 cases) were treated and resolved with placement of an endoscopic stent while the low-grade leak resolved spontaneously. *Candida tropicalis* infection of the peritoneal fluid was identified in one patient and controlled with antifungal therapy.

Three patients underwent surgical re-operation. Patient 6 required surgical haemostasis of the wound 5 days after ALPPS-2. Patient 7 underwent exploratory laparotomy 4 days after ALPPS-1 to verify the viability of the liver in the presence of a peak of transaminase (AST 2998 U/L; ALT: 3097 U/L). Liver biopsy showed severe necrosis of the right liver. Cytolysis gradually diminished over the following days. Patient 9 required re-operation for haemoperitoneum 2 days after ALPPS-2.

After a mean follow-up of 17.1±8.5 months, the overall survival was 89% at 3-6-12 months. Recurrence-free survival was 100%, 87.5% and 75% at 3-6-12 months respectively. Four patients (case 2, 3, 5 and 8) developed a tumor recurrence after 14.7, 19.6, 3.2 and 6.3 months, respectively but they are currently alive.

The characteristics of the 9 patients who underwent the ALPPS procedure are described in Table 6.

**Table 6:** Patient characteristics.

Pt No	Gender	Age	Tumor	Number of nodules	Total nodules diameter (cm)	FLR (mL) before and after ALPPS1	FLR (%) before and after ALPPS1	FLR Increase(%)	Liver Resection (segments)	Hospital status	Status	Follow-up (months)
1	F	54	pCCA	1	NA	318/665	24/30	109	1-4-5-6-7-8	29	Alive	29,53
2	F	66	iCCA	2	10	393/566	27/34	44	1-4-5-6-7-8	27	Alive	24,23
3	F	69	CRLM	1	11	174/348	22/33	100	1-4-5-6-7-8	16	Alive	23,57
4	F	60	pCCA	1	NA	208/540	15/36	160	1-4-5-6-7-8	15	Alive	19,89
5	F	49	HCC	1	14	552/684	25/24	24	4-5-6-7-8	23	Alive	17,13
6	F	73	pCCA	1	NA	192/395	16/24	105	1-4-5-6-7-8	15	Dead	0,69
7	M	53	CRLM	4	9.5	194/502	14/35	159	4b-5-6-7-8	24	Alive	13,21
8	M	62	CRLM	1	5	304/632	18/32	108	4-5-6-7-8	26	Alive	13,21
9	F	54	CRLM	4	9.7	270/421	29/42	56	4-5-6-7-8	56	Alive	12,26

pCCA = perihilar cholangiocarcinoma; iCCA = Intrahepatic cholangiocarcinoma; CRLM: Colorectal liver metastases; HCC = Hepatocellular carcinoma. NA: not applicable.

### Systematic Review

#### Study selection

**Fig 4:**
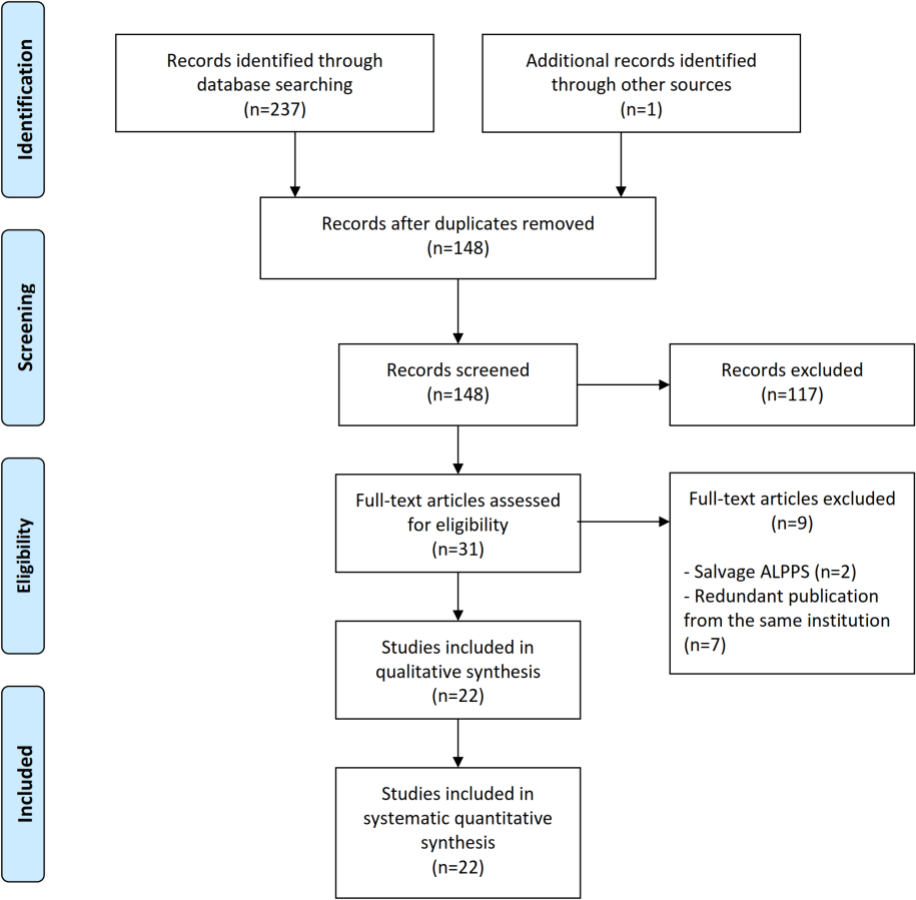
Study selection.

The literature search yielded 238 articles; after the removal of duplicates, 148 titles and abstracts were reviewed (Fig 4). Of these, 117 papers were excluded for the following reasons: 45 papers not concerning the ALPPS procedure, 53 letters to the editor, comments or case reports, 12 review articles, and 7 papers in languages other than English. Thirty-one articles were selected for full-text review; of these, 7[13–19] were excluded because they were redundant from the same institution and two[20, 21] because they described a salvage ALPPS procedure.

Finally, a total of 22 articles[4, 22–42] published between 2012 and 2014 fulfilled the selection criteria and were included in this systematic review. The most common bias found was related to retrospective analyses and lack of a control group in all studies. Only one paper retrospectively compared ALPPS to the PVO procedure[34], while no randomized studies are described; three papers reported multicentric data[4, 34, 35]. All these studies included a total of 335 adult patients. One paper described the early results of the multicentric international ALPPS registry that includes 202 patients from 41 centres[38].

Several variations of the classical ALPPS were described, namely:

- Associating Liver Tourniquet and Partial ligation for Staged hepatectomy (ALTPS)[33]. The authors propose to avoid the partition of the liver in the first surgery; the vascular flow between the two hemilivers is occluded by a tourniquet that is functionally tightened around the Cantlie’s line. The absence of flow between segments II-III and IV is then confirmed by ultrasound examination. The results with this method are not functionally different from the traditional method, and the authors report less blood loss and shorter operative time.
- "Left ALPPS": ligation of the left portal vein, multiple resections on the right hemiliver and splitting along the main portal fissure, proposed for bilateral liver metastases where a left hepatectomy is required in addition to multiple resections on the right liver[43].
- "Salvage ALPPS": simple splitting of the liver along the main portal fissure several months after a radiological portal vein embolization that did not allow satisfactory liver hypertrophy[20, 23, 43, 44].
- "Right ALPPS": ligation of the posterolateral branch of the right portal vein, left lateral sectionectomy, multiple resections on the right anterior and left medial section and splitting along the right portal fissure; this technique was proposed to deal with tumors that require simultaneous resection of the lateral segments of the left and right liver[43].
- Laparoscopic ALPPS procedures[24, 45, 46].
- “Partial ALPPS” has recently been described preliminarily: in this technique the parenchymal transection during the first step of the procedure is not complete but carried out to obtain a partition of at least 50% of the liver along the resection plane. The authors report lower mortality and rate of complications with this technique as compared to the conventional ALPPS, with a similar degree of hypertrophy of the FLR[47].

Outcomes of interest of each single study are summarized in Tables 7 and 8. Excluding data from ALPPS registry, to avoid patient overlap, the main indication of ALPPS was CRLMs (68.7%), whereas cholangiocarcinoma was 14%.

**Table 7:** Demographics, indication for surgery, radiologic data and outcome in systematic review.

	Pts	M/F	Age (years)	CRLM	CCA	FLR (%) increase	R0	Morbidity	Mortality	Interval between 2 steps (days)	Hospital duration(days)
**Schnitzbauer, 2012**[4]	25	14/11	63 (32–75)	11 (44%)	4 (16%)	74 (21–192)	96%	64%	12%	NA	NA
**Alvarez, 2013**[22]	15	9/6	54	10 (66.7%)	1 (6.7%)	78.4	100%	53%	0%	NA	19
**Brustia, 2013**[24]	6	NA	NA	4 (66.7%)	0	43	NA	NA	NA	8	NA
**Björnsson, 2013**[23]	2	2/0	77–80	1 (50%)	0	106	100%	0%	NA	9–7	10
**Torres, 2013**[35]	39	22/17	57.3 (20–83)	32 (82%)	3 (7.7%)	83 (47–211.9)	NA	59.0%	12.8	14.1 (5–30)	17.8 (13–40)
**Knoefel, 2013**[28]	7	NA	NA	4 (57%)	NA	63 (29)	NA	71.4%	NA	6 (4–8)	NA
**Ielpo, 2013**[27]	6	2/4	59.2 (56–63)	5 (83.3%)	1 (16.7%)	110	NA	100%	16.7%	16 (12–21)	34.2
**Li, 2013**[29]	9	4/5	NA	3 (33.3%)	6 (66.7%)	87.2 (23.8–161)	NA	NA	NA	13 (9–18)	NA
**Hernandez, 2014**[26]	14	9/5	57 (31–66)	14 (100%)	0	93±28	86%	36%	NA	8±1	23±12
**Oldhafer, 2014**[31]	7	4/3	65.7	7 (100%)	0	65 (16–97)	100%	NA	NA	13 (10–40)	NA
**Troja, 2014**[36]	5	4/1	59.6	2 (40%)	1 (20%)	NA	100%	100%	20%	NA	56.7
**Robles, 2014**[33]	22	17/5	65 (35–80)	17 (77.3%)	0	61 (33–189)	NA	63.6%	9.1%	11 (8–28)	16 (12–28)
**Schadde, 2014**[34]	48	NA	NA	26 (54.2%)	10 (20.8%)	77	NA	NA	15%	NA	NA
**Nadalin, 2014**[30]	15	7/8	67 (43–80)	5 (33.3%)	9 (60%)	87.2 (23.8−161)	86.7%	66.7%	28.7%	13 (9−18)	NA
**Vennarecci, 2014**[37]	3	NA	58.3	0	0	31.7	NA	100%	0%	NA	21
**Ratti, 2014**[32]	6	4/2	62.5	3 (50%)	2 (33.3%)	62.2	100%	66.7%	0%	NA	NA
**De Carlis, 2014**[25]	3	2/1	66.7 (59–76)	2 (66.7%)	1 (33.3%)	57.4 (33.2–79.1)	NA	NA	NA	NA	NA
**Croome, 2015**[39]	15	11/4	55.9±12.1	14 (93%)	0	36.1±6.4	NA	NA	0%	7.8±1.1	NA
**Herman, 2015**[40]	7	5/2	52.4±7.39	6 (85.7%)	1	68.7±15.9	NA	57.1%	0%	8.1±2.6	20.4 (13–38)
**Tanaka, 2015**[41]	10	7 (64%)	68 (50–78)	10 (91%)	0	52.9	100%	7 (63.6%)	1 (9%)	NA	11.0 (8–54)
**Truant, 2015**[42]	62	45 (72.6%)	59.1±9.3	50 (80.6%)	3 (4.8%)	48.6 (-15.3–192)	NA	50 (80.6%)	8 (12.9%)	7.8±4.5	29.2±24.1
**Present series, 2015**	9	2/7	60±8	4 (44.4%)	4 (44.4%)	96 (44–160)	100%	67%	11.1%	11 (7–15)	22 (13–55)
**ALPPS registry**[38]	202	121/81	60	141 (70%)	19 (9.4%)	80	NA	NA	9%	NA	20

NA: not available.

**Table 8:** Results of systematic review: intraoperative data.

	ALPPS-1			ALPPS-2	
	Surgical time (min)	Blood loss (ml)	Pringle manoeuvre	Surgical time (min)	Blood loss (ml)
**Schnitzbauer, 2012[4]**	210 (157–500)	NA	24%	152 (64–364)	NA
**Alvarez, 2013[22]**	326	NA	33.3%	139	NA
**Brustia, 2013[24]**	277	NA	NA	216	NA
**Björnsson, 2013[23]**	295–164	2000–300	NA	90–55	NA
**Vennarecci, 2014[37]**	300	150	33.3%	180	50
**Hernandez, 2014[26]**	385±59	725±85	NA	144±41	178±80
**Robles, 2014[33]**	125 (120–240)	100 (0–900)	NA	150 (90–330)	200 (0–1500)
**Present series, 2014**	429±113 (300–640)	416±400	33.3%	198±59	222 (0–600)
**ALPPS Registry[38]**	327±119	NA	NA	156±75	NA

NA: not available.

The median age of the patients who underwent ALPPS ranged between 52.4 and 68 years in the various reports with the youngest patient of 20 and the oldest of 83 years. The median preoperative FLR ranged between 19 and 27%; after a median interval of 6–16 days, all the studies reported an outstanding increase in the volume of the FLR that ranged between 36.1 and 110%. ALPPS procedure achieved a rate of R0 surgical margins that ranged between 86 and 100%. The morbidity was particularly high in all the series (from 36 to 100% of the cases in the single series). Although a few Centers reported mortality-free experiences, the largest series available (ALLPS registry) recorded a 9% postoperative mortality. Median hospital stay ranged between 11 and 56.7 days.

No intention-to-treat analysis was available that reported the number of planned ALPPS procedures that were eventually not performed due to intraoperative findings during the first step.

### Discussion

The ALPPS technique has recently been described as an alternative to preoperative embolization or ligation of the right branch of the portal vein when extended liver resections are required in the presence of insufficient FLR. In fact, the strategy of PVO has some important limitations: 3 to 8 weeks are necessary to develop sufficient hypertrophy of the FLR[2]; therefore, the risk of neoplastic progression between the two interventions is high. Additionally, PVO failed to induce compensatory hypertrophy in 14% of the cases[48], while obviously it cannot be used in the presence of tumor invasion of the right branch of the portal vein[37]. In addition, the rates of hypertrophy that can be achieved with PVO range between 10 and 46%[2, 49, 50]. Globally, PVO fail to allow resectability in up to 33%[20].

The ALPPS procedure seems to overcome these limitations as it rapidly induces a consistently more pronounced hypertrophy of the FLR compared with conventional procedures. The presumed mechanism of the quick hypertrophy of the FLR is the interruption of the cross portal circulation between the 2 hemilivers that allows a complete diversion of the portal flow to the FLR[22]. In addition, the diseased hemiliver acts as a transitory auxiliary liver that assists the growing FLR in metabolic, synthetic and detoxifying functions for the first and critical week after liver partition.

A peculiarity of our series is that the majority of the patients were treated for tumors different from CRLM which was instead the most common indication in the ALPPS registry (70% of the cases) and was associated with better survival. Besides, 3 of our patients had pCCA, a disease that is considered to be at greater risk of complications and perioperative mortality; in the ALPPS registry there were only 11 (5.4%) cases of pCCA and a 27% postoperative mortality was recorded in these patients while in the single-Institution series that included the highest number of pCCAs (5 patients) the operative mortality was 60%[30, 38].

A recent comprehensive meta-analysis on the use of PVE to increase operability of cholangiocellular carcinoma has shown that this is effective when the mean initial FLR is 33.8%; of 836 cases included in that analysis, no one had a FLR below 30%. The efficacy of PVE in cases similar to those reported in the present series, whose FLR was well below 30%, is therefore far from being demonstrated[51].

Although successful ALPPS procedures in patients with hepatocellular carcinoma on cirrhosis have been reported, in our policy chronic liver disease is currently considered a contraindication to surgery [37, 52, 53]. We believe that when the liver is affected by a chronic disease, it is hazardous to plan extensive demolition relying on the functional reserve as assessed by conventional staging systems such as Child-Pugh and MELD score or by functional tests such as indocyanine green test. The only postoperative death that was observed in our series was due to liver failure and this occurred in the only patient who had liver fibrosis: this evidence seems to support our policy towards ALPPS in chronic liver disease.

There was a large variability in the increase of FLR, from 24% to 159% and the lowest increase in the liver volume was observed in the only patient who did not survive. Alongside the increase of FLR after ALPPS-1, in four cases an increase in the volume of the liver tumors was observed. This interesting finding may be the result of the arterialization of the right hemiliver that follows the ligation of the right branch of the portal vein. In contrast to the normal parenchyma, neoplastic lesions base their blood supply exclusively on the arterial inflow; consequently, arterialization of the tumor lesions in a deportalized right liver can produce an increased tumor volume.

In our experience, sufficient hypertrophy of FLR was obtained in 10.8 days: this time span is significantly lower than the average time required when using PVO (40 days), minimizing the risk of progression of the tumor between the two steps of the procedure [3, 17].

Our experience confirms that a high incidence of postoperative complications can be expected after ALPPS: 67% of our patients experienced complications that were grade 3 or above in 44% of the cases. The main complications reported in the literature were biliary fistulas, sepsis and infection. In our series, the most frequent complication was biliary fistula (3 cases). These data are similar to those reported in other series, however, we treated many more patients with CCA and, in particular, pCCA; consequently, an even higher risk of biliary complications might have been expected.

The presence of a biliary stent placed to treat the biliary obstruction caused by pCCA was a predisposing factor for intraabdominal sepsis: this latter complication was observed in 2 of 2 patients with preoperative stenting and in 1 of 7 patients with no preoperative stenting.

In our series, all liver resections were oncologically radical, consistent with what was reported in most reports from the literature[22, 23, 31, 32, 36].

Overall, the one-year survival in our study was 89%. This result is relevant because several cases of biliary tumors were offered treatment in contrast to the majority of the other series where biliary cancer was considered a contraindication to ALPPS[30]. As the only postoperative death in our series was observed in a 73-year-old patient, we now limit the procedure to patients younger than 70 years. Age >60 was indeed associated with poorer survival in the report from the ALLPS Registry[38].

In the patient who did not survive, several risk factors for postoperative complications (presence of a biliary stent) and poor outcome (age >70 and presence of fibrosis at liver histology) were concomitant. Sepsis that developed after step 1 also contributed to impair the hypertrophy of the FLR in this patient and the attempt to remove the source of the sepsis by completing the ALPPS was unsuccessful: we would therefore recommend to focus on the treatment of the sepsis and delay the completion of the ALPPS in similar circumstances.

Based on our experience, we believe that ALPPS should be indicated in patient of less than 70 years of age with no major comorbidities and no chronic liver disease as confirmed by the absence of fibrosis at liver biopsy; liver biopsy should be performed before proceeding to liver partition. In view of the morbidity and mortality that are associated with ALPPS, this procedure should be offered in first instance only when the intrahepatic diffusion of the tumor makes it hazardous to delay its excision in wait for the hypertrophy of the FLR to be achieved through PVO techniques.

In our centre, we perform the conventional ALPPS technique. Several variants of the conventional technique have been described: some of these techniques such as “left” and “right” ALPPS can be useful in particular anatomical distributions of the tumors, mainly in case of CRLMs. Among the other variations proposed, “partial ALPPS”, seems to be able to induce an hypertrophy of the FLR similar to that observed in the conventional ALPPS and reduce postoperative mortality and complications by avoiding the complete partition of the liver during step 1; if these findings were confirmed on larger numbers (only 6 cases of partial ALPPS have been reported to date), partial ALPPS could well become the technique of choice.

The latest evidence suggests that the use of a plastic bag to separate the two hemi-livers should be avoided because the presence of foreign material in the abdomen seems to correlate with the development of infectious events and adhesions[46, 54]. Furthermore, the presence of plastic material necessarily requires a second surgery, even when the second step of ALPPS should eventually become contraindicated. In our experience, we now routinely utilize TachoSil.

The main limitations of this study were the low sample size and lack of a comparison with alternative techniques, such as PVE or PVL. Future studies will need to compare this technique with alternative ones, although the excellent results reported in the literature might pose ethical issues. A prospective, randomized comparison among the PVE, PVL and ALPPS techniques is ongoing presently with several participating centres worldwide.

The systematic review of the literature has shown a growing worldwide interest in this novel surgical technique. The results of the ALPPS that are reported in the literature are promising however, many of the published articles lack of a detailed report of the postoperative morbidity according with Dindo-Clavien classification and long-terms results of the procedure need to be further analysed and validated through a prospective, multicentre randomized study[55].

In conclusion, our initial experience with liver resections using the ALPPS technique showed that the procedure is effective in patients with insufficient FLR. The morbidity was high in accordance with the data from the literature, and we had one mortality. However, it has to be noted that all the patients in this series had diseases with an ominous prognosis with no alternative effective treatment available.

## Supporting Information

S1 Appendix PRISMA Checklist. (DOC)

S1 Dataset Volumetry dataset. (XLSX)

## Abbreviations

FLR: future liver remnant
PVE: portal embolization
PVL: portal vein ligation
PVO: portal vein occlusion
ALPPS: Associating Liver Partition and Portal vein Ligation for Staged hepatectomy
TLV: total liver volume
TuV: tumor volume
pCCA: perihilar cholangiocarcinoma
iCCA: intrahepatic cholangiocarcinoma
HCC: hepatocellular carcinoma
CEA: carcinoembryonic antigen
α-FP: serum alphafetoprotein
ALTPS: Associating Liver Tourniquet and Partial ligation for Staged hepatectomy

## References

[1] Adams RB, Aloia TA, Loyer E, Pawlik TM, Taouli B, Vauthey JN, et al Selection for hepatic resection of colorectal liver metastases: expert consensus statement. HPB: the official journal of the International Hepato Pancreato Biliary Association. 2013;15(2):91–103. 10.1111/j.1477-2574.2012.00557.x 23297719 PMC3719914

[2] Farges O, Belghiti J, Kianmanesh R, Regimbeau JM, Santoro R, Vilgrain V, et al Portal vein embolization before right hepatectomy: prospective clinical trial. Annals of surgery. 2003;237(2):208–17. 10.1097/01.SLA.0000048447.16651.7B 12560779 PMC1522143

[3] Kokudo N, Tada K, Seki M, Ohta H, Azekura K, Ueno M, et al Proliferative activity of intrahepatic colorectal metastases after preoperative hemihepatic portal vein embolization. Hepatology. 2001;34(2):267–72. 10.1053/jhep.2001.26513 . 11481611

[4] Schnitzbauer AA, Lang SA, Goessmann H, Nadalin S, Baumgart J, Farkas SA, et al Right portal vein ligation combined with in situ splitting induces rapid left lateral liver lobe hypertrophy enabling 2-staged extended right hepatic resection in small-for-size settings. Annals of surgery. 2012;255(3):405–14. 10.1097/SLA.0b013e31824856f5 22330038

[5] Dindo D, Demartines N, Clavien PA. Classification of surgical complications: a new proposal with evaluation in a cohort of 6336 patients and results of a survey. Annals of surgery. 2004;240(2):205–13. 15273542 10.1097/01.sla.0000133083.54934.ae PMC1360123

[6] Schindl MJ, Redhead DN, Fearon KC, Garden OJ, Wigmore SJ, Edinburgh Liver S, et al The value of residual liver volume as a predictor of hepatic dysfunction and infection after major liver resection. Gut. 2005;54(2):289–96. 10.1136/gut.2004.046524 15647196 PMC1774834

[7] Reiner CS, Karlo C, Petrowsky H, Marincek B, Weishaupt D, Frauenfelder T. Preoperative liver volumetry: how does the slice thickness influence the multidetector computed tomography- and magnetic resonance-liver volume measurements? Journal of computer assisted tomography. 2009;33(3):390–7. 10.1097/RCT.0b013e3181806c29 . 19478632

[8] Zappa M, Dondero F, Sibert A, Vullierme MP, Belghiti J, Vilgrain V. Liver regeneration at day 7 after right hepatectomy: global and segmental volumetric analysis by using CT. Radiology. 2009;252(2):426–32. 10.1148/radiol.2522080922 . 19703882

[9] Donati M, Basile F, Oldhafer KJ. Present status and future perspectives of ALPPS (associating liver partition and portal vein ligation for staged hepatectomy). Future Oncol. 2015;11(16):2255–8. 10.2217/fon.15.145 . 26260803

[10] Jacobson BF, Louw S, Buller H, Mer M, de Jong PR, Rowji P, et al Venous thromboembolism: prophylactic and therapeutic practice guideline. South African medical journal = Suid-Afrikaanse tydskrif vir geneeskunde. 2013;103(4 Pt 2):261–7. 10.7196/samj.6706 . 23547704

[11] Balzan S, Belghiti J, Farges O, Ogata S, Sauvanet A, Delefosse D, et al The "50–50 criteria" on postoperative day 5: an accurate predictor of liver failure and death after hepatectomy. Annals of surgery. 2005;242(6):824–8, discussion 8–9. 16327492 10.1097/01.sla.0000189131.90876.9e PMC1409891

[12] Liberati A, Altman DG, Tetzlaff J, Mulrow C, Gotzsche PC, Ioannidis JP, et al The PRISMA statement for reporting systematic reviews and meta-analyses of studies that evaluate health care interventions: explanation and elaboration. PLoS medicine. 2009;6(7):e1000100 10.1371/journal.pmed.1000100 19621070 PMC2707010

[13] De Santibañes E, Alvarez FA, Ardiles V. How to avoid postoperative liver failure: A novel method. World journal of surgery. 2012;36(1):125–8. 10.1007/s00268-011-1331-0 22045448

[14] Ielpo B, Quijano Y, Vicente E. Pearls and pitfalls on ALPPS procedure: new complications in a new technique. Updates in surgery. 2014;66(2):159–61. Epub 2014/03/04. 10.1007/s13304-014-0249-0 . 24584837

[15] Robles Campos R, Parrilla Paricio P, Lopez Conesa A, Brusadin R, Lopez Lopez V, Jimeno Grino P, et al [A new surgical technique for extended right hepatectomy: tourniquet in the umbilical fissure and right portal vein occlusion (ALTPS). Clinical case]. Cirugia espanola. 2013;91(10):633–7. Epub 2013/11/20. 10.1016/j.ciresp.2013.09.004 . 24246509

[16] Sala S, Ardiles V, Ulla M, Alvarez F, Pekolj J, De Santibañes E. Our initial experience with ALPPS technique: Encouraging results. Updates in surgery. 2012;64(3):167–72. 10.1007/s13304-012-0175-y 22903531

[17] Torres OJ, Moraes-Junior JM, Lima e Lima NC, Moraes AM. Associating liver partition and portal vein ligation for staged hepatectomy (ALPPS): a new approach in liver resections. Arquivos brasileiros de cirurgia digestiva: ABCD = Brazilian archives of digestive surgery. 2012;25(4):290–2. Epub 2013/02/16. . 23411931 10.1590/s0102-67202012000400015

[18] Ulla M, Ardiles V, Levy-Yeyati E, Alvarez FA, Spina JC, Garcia-Mońaco RD, et al New surgical strategy to induce liver hypertrophy: Role of MDCT-volumetry to monitor and predict liver growth. Hepato-gastroenterology. 2013;60(121):337–42. 23169065 10.5754/hge12717

[19] Vennarecci G, Laurenzi A, Santoro R, Colasanti M, Lepiane P, Ettorre GM. The ALPPS procedure: a surgical option for hepatocellular carcinoma with major vascular invasion. World journal of surgery. 2014;38(6):1498–503. Epub 2013/10/23. 10.1007/s00268-013-2296-y . 24146197

[20] Tschuor C, Croome KP, Sergeant G, Cano V, Schadde E, Ardiles V, et al Salvage parenchymal liver transection for patients with insufficient volume increase after portal vein occlusion—an extension of the ALPPS approach. European journal of surgical oncology: the journal of the European Society of Surgical Oncology and the British Association of Surgical Oncology. 2013;39(11):1230–5. Epub 2013/09/03. 10.1016/j.ejso.2013.08.009 . 23994139

[21] Vyas SJ, Davies N, Grant L, Imber CJ, Sharma D, Davidson BR, et al Failure of Portal Venous Embolization. ALPPS as Salvage Enabling Successful Resection of Bilobar Liver Metastases. Journal of gastrointestinal cancer. 2014 Epub 2014/08/02. 10.1007/s12029-014-9643-6 . 25081490

[22] Alvarez FA, Ardiles V, Sanchez Claria R, Pekolj J, de Santibañes E. Associating Liver Partition and Portal Vein Ligation for Staged Hepatectomy (ALPPS): Tips and Tricks. Journal of Gastrointestinal Surgery. 2013;17(4):814–21. 10.1007/s11605-012-2092-2 23188224

[23] Bjornsson B, Gasslander T, Sandstrom P. In situ split of the liver when portal venous embolization fails to induce hypertrophy: a report of two cases. Case reports in surgery. 2013;2013:238675 Epub 2014/01/03. 10.1155/2013/238675 24383035 PMC3871496

[24] Brustia R, Scatton O, Perdigao F, El-Mouhadi S, Cauchy F, Soubrane O. Vessel identifications tags for open or laparoscopic associating liver partition and portal vein ligation for staged hepatectomy. Journal of the American College of Surgeons. 2013;217(6):e51–5. Epub 2013/11/20. 10.1016/j.jamcollsurg.2013.08.020 . 24246632

[25] De Carlis L, Sguinzi R, De Carlis R, Di Sandro S, Mangoni J, Aseni P, et al Residual right portal branch flow after first-step ALPPS: Artifact or homeostatic response? Hepato-gastroenterology. 2014;61(134):1712–6. 25436367

[26] Hernandez-Alejandro R, Bertens KA, Pineda-Solis K, Croome KP. Can we improve the morbidity and mortality associated with the associating liver partition with portal vein ligation for staged hepatectomy (ALPPS) procedure in the management of colorectal liver metastases? Surgery. 2014 Epub 2014/10/06. 10.1016/j.surg.2014.08.041 . 25282528

[27] Ielpo B, Caruso R, Ferri V, Quijano Y, Duran H, Diaz E, et al ALPPS procedure: our experience and state of the art. Hepato-gastroenterology. 2013;60(128):2069–75. Epub 2014/04/11. . 24719949

[28] Knoefel WT, Gabor I, Rehders A, Alexander A, Krausch M, Schulte am Esch J, et al In situ liver transection with portal vein ligation for rapid growth of the future liver remnant in two-stage liver resection. The British journal of surgery. 2013;100(3):388–94. 10.1002/bjs.8955 . 23124776

[29] Li J, Girotti P, Königsrainer I, Ladurner R, Königsrainer A, Nadalin S. ALPPS in Right Trisectionectomy: A Safe Procedure to Avoid Postoperative Liver Failure? Journal of Gastrointestinal Surgery. 2013;17(5):956–61. 10.1007/s11605-012-2132-y 23288719

[30] Nadalin S, Capobianco I, Li J, Girotti P, Königsrainer I, Königsrainer A. Indications and limits for associating liver partition and portal vein ligation for staged hepatectomy (ALPPS)lessons learned from 15 cases at a single centre. Zeitschrift fur Gastroenterologie. 2014;52(1):35–42. 10.1055/s-0033-1356364 24420797

[31] Oldhafer KJ, Donati M, Jenner RM, Stang A, Stavrou GA. ALPPS for patients with colorectal liver metastases: effective liver hypertrophy, but early tumor recurrence. World journal of surgery. 2014;38(6):1504–9. Epub 2013/12/12. 10.1007/s00268-013-2401-2 . 24326456

[32] Ratti F, Cipriani F, Gagliano A, Catena M, Paganelli M, Aldrighetti L. Defining indications to ALPPS procedure: technical aspects and open issues. Updates in surgery. 2014;66(1):41–9. Epub 2013/12/18. 10.1007/s13304-013-0243-y . 24343420

[33] Robles R, Parrilla P, Lõpez-Conesa A, Brusadin R, De La Peña J, Fuster M, et al Tourniquet modification of the associating liver partition and portal ligation for staged hepatectomy procedure. British Journal of Surgery. 2014;101(9):1129–34. 10.1002/bjs.9547 24947768

[34] Schadde E, Ardiles V, Slankamenac K, Tschuor C, Sergeant G, Amacker N, et al ALPPS offers a better chance of complete resection in patients with primarily unresectable liver tumors compared with conventional-staged hepatectomies: Results of a multicenter analysis. World journal of surgery. 2014;38(6):1510–9. 10.1007/s00268-014-2513-3 24748319

[35] Torres OJ, Fernandes ES, Oliveira CV, Lima CX, Waechter FL, Moraes-Junior JM, et al Associating liver partition and portal vein ligation for staged hepatectomy (ALPPS): the Brazilian experience. Arquivos brasileiros de cirurgia digestiva: ABCD = Brazilian archives of digestive surgery. 2013;26(1):40–3. 23702869 10.1590/s0102-67202013000100009

[36] Troja A, Khatib-Chahidi K, El-Sourani N, Antolovic D, Raab HR. ALPPS and similar resection procedures in treating extensive hepatic metastases: our own experiences and critical discussion. International journal of surgery (London, England). 2014;12(9):1020–2. Epub 2014/07/22. 10.1016/j.ijsu.2014.07.006 . 25043935

[37] Vennarecci G, Laurenzi A, Levi Sandri GB, Busi Rizzi E, Cristofaro M, Montalbano M, et al The ALPPS procedure for hepatocellular carcinoma. European journal of surgical oncology: the journal of the European Society of Surgical Oncology and the British Association of Surgical Oncology. 2014;40(8):982–8. Epub 2014/04/29. 10.1016/j.ejso.2014.04.002 . 24767805

[38] Schadde E, Ardiles V, Robles-Campos R, Malago M, Machado M, Hernandez-Alejandro R, et al Early Survival and Safety of ALPPS: First Report of the International ALPPS Registry. Annals of surgery. 2014;260(5):829–38. 10.1097/SLA.0000000000000947 . 25379854

[39] Croome KP, Hernandez-Alejandro R, Parker M, Heimbach J, Rosen C, Nagorney DM. Is the liver kinetic growth rate in ALPPS unprecedented when compared with PVE and living donor liver transplant? A multicentre analysis. HPB: the official journal of the International Hepato Pancreato Biliary Association. 2015 Epub 2015/03/03. 10.1111/hpb.12386 . 25728543 PMC4430776

[40] Herman P, Krüger JAP, Perini MV, Coelho FF, Cecconello I. High Mortality Rates After ALPPS: the Devil Is the Indication. Journal of gastrointestinal cancer. 2015 10.1007/s12029-015-9691-6 25682120

[41] Tanaka K, Matsuo K, Murakami T, Kawaguchi D, Hiroshima Y, Koda K, et al Associating liver partition and portal vein ligation for staged hepatectomy (ALPPS): Short-term outcome, functional changes in the future liver remnant, and tumor growth activity. European journal of surgical oncology: the journal of the European Society of Surgical Oncology and the British Association of Surgical Oncology. 2015;41(4):506–12. Epub 2015/02/24. 10.1016/j.ejso.2015.01.031 . 25704556

[42] Truant S, Scatton O, Dokmak S, Regimbeau JM, Lucidi V, Laurent A, et al Associating liver partition and portal vein ligation for staged hepatectomy (ALPPS): Impact of the inter-stages course on morbi-mortality and implications for management. European journal of surgical oncology: the journal of the European Society of Surgical Oncology and the British Association of Surgical Oncology. 2015;41(5):674–82. Epub 2015/01/30. 10.1016/j.ejso.2015.01.004 . 25630689

[43] Gauzolino R, Castagnet M, Blanleuil ML, Richer JP. The ALPPS technique for bilateral colorectal metastases: three "variations on a theme". Updates in surgery. 2013;65(2):141–8. Epub 2013/05/22. 10.1007/s13304-013-0214-3 . 23690242

[44] Jackson T, Siegel KA, Siegel CT. Rescue ALPPS: Intraoperative Conversion to ALPPS during Synchronous Resection of Rectal Cancer and Liver Metastasis. Case reports in surgery. 2014;2014:487852 Epub 2014/12/17. 10.1155/2014/487852 25506458 PMC4259134

[45] Kilburn DJ, Chiow AKH, Lewin J, Kienzle N, Cavallucci DJ, Bryant R, et al Laparoscopic approach to a planned two-stage hepatectomy for bilobar colorectal liver metastases. ANZ journal of surgery. 2014. 10.1111/ans.12748 24990234

[46] MacHado MAC, Makdissi FF, Surjan RC. Totally laparoscopic ALPPS is feasible and may be worthwhile. Annals of surgery. 2012;256(3). 10.1097/SLA.0b013e318265ff2e 22842130

[47] Petrowsky H, Gyori G, de Oliveira M, Lesurtel M, Clavien PA. Is Partial-ALPPS Safer Than ALPPS? A Single-center Experience. Annals of surgery. 2015;261(4):e90–2. Epub 2015/02/24. 10.1097/sla.0000000000001087 . 25706390

[48] Zhang GQ, Zhang ZW, Lau WY, Chen XP. Associating liver partition and portal vein ligation for staged hepatectomy (ALPPS): a new strategy to increase resectability in liver surgery. International journal of surgery (London, England). 2014;12(5):437–41. Epub 2014/04/08. 10.1016/j.ijsu.2014.03.009 . 24704086

[49] Liu H, Zhu S. Present status and future perspectives of preoperative portal vein embolization. American journal of surgery. 2009;197(5):686–90. 10.1016/j.amjsurg.2008.04.022 . 19249737

[50] Aussilhou B, Lesurtel M, Sauvanet A, Farges O, Dokmak S, Goasguen N, et al Right portal vein ligation is as efficient as portal vein embolization to induce hypertrophy of the left liver remnant. Journal of gastrointestinal surgery: official journal of the Society for Surgery of the Alimentary Tract. 2008;12(2):297–303. Epub 2007/12/07. 10.1007/s11605-007-0410-x . 18060468

[51] Higuchi R, Yamamoto M. Indications for portal vein embolization in perihilar cholangiocarcinoma. Journal of hepato-biliary-pancreatic sciences. 2014;21(8):542–9. 10.1002/jhbp.77 . 24520045

[52] Cavaness KM, Doyle MBM, Lin Y, Maynard E, Chapman WC. Using ALPPS to Induce Rapid Liver Hypertrophy in a Patient with Hepatic Fibrosis and Portal Vein Thrombosis. Journal of Gastrointestinal Surgery. 2013;17(1):207–12. 10.1007/s11605-012-2029-9 22996934

[53] Ding X, Carrasco-Avino G, Thung SN, Roayaie S. A two-step right hepatic lobectomy with portal vein ligation for large hepatocellular carcinoma: rapid induction of left-lobe regeneration and clinicopathologic correlation. Seminars in liver disease. 2013;33(3):293–7. Epub 2013/08/15. 10.1055/s-0033-1351786 . 23943109

[54] Chan A, Chung PHY, Poon RTP. Little girl who conquered the "ALPPS". World Journal of Gastroenterology. 2014;(29):10208–11. 10.3748/wjg.v20.i29.10208 25110450 PMC4123352

[55] Schadde E, Schnitzbauer AA, Tschuor C, Raptis DA, Bechstein WO, Clavien PA. Systematic Review and Meta-Analysis of Feasibility, Safety, and Efficacy of a Novel Procedure: Associating Liver Partition and Portal Vein Ligation for Staged Hepatectomy. Annals of surgical oncology. 2015;22(9):3109–20. Epub 2014/12/03. 10.1245/s10434-014-4213-5 . 25448799

